# Nanogels—Innovative Drug Carriers for Overcoming Biological Membranes

**DOI:** 10.3390/gels11020124

**Published:** 2025-02-08

**Authors:** Lyubomira Radeva, Krassimira Yoncheva

**Affiliations:** Faculty of Pharmacy, Medical University of Sofia, 1000 Sofia, Bulgaria; l.radeva@pharmfac.mu-sofia.bg

**Keywords:** nanogels, polymers, drug loading, drug release, biological membranes

## Abstract

Nanogels are promising drug delivery systems since they possess undeniable advantages such as high loading capacity for hydrophilic and hydrophobic drugs, stabilization of sensitive drugs, biocompatibility, and biodegradability. The present review summarizes experimental studies related to carriers, drug loading, and membrane transport of nanogels. In particular, the review discusses the properties, advantages, and limitations of polymeric carriers with respect to the behavior of the prepared nanogels in in vivo conditions. The potential of nanogel systems for encapsulation of hydrophilic or hydrophobic drugs and the mechanisms of loading and drug release are also emphasized. Moreover, the challenges related to nanogel transport through the barriers presented in parenteral, oral, ocular, nasal, and dermal routes of administration are also considered.

## 1. Introduction

**Figure 1:**
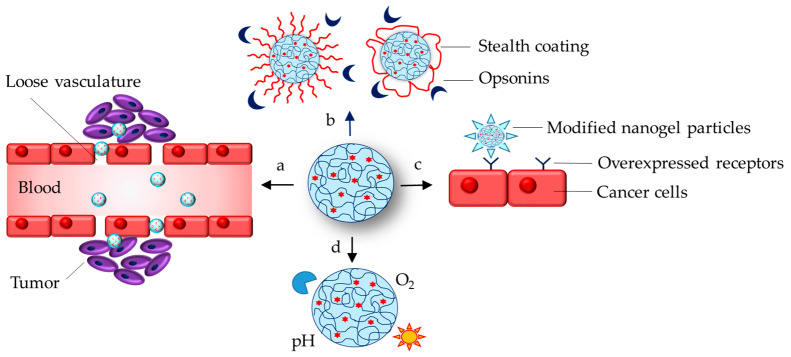
Schematic presentation of advantages of nanogel drug delivery systems related to their nanoparticle nature: enhanced permeation and retention of nanogel particles into tumor tissue (EPR effect) due to their small size (**a**), avoidance of opsonisation by pegylated surface with different conformation of PEG chains (**b**), receptor-mediated transport of nanogel particles modified with targeting ligands (**c**), and protection of the encapsulated active molecules against different in vitro (oxygen and light) and in vivo (enzymes and pH) inappropriate conditions (**d**).

Nanogels are a type of nanoparticles that have a three-dimensional cross-linked network of polymer chains. Thus, the nanogels combine the properties of nanoparticles (e.g., nanosize and colloidal stability) and those of hydrogels (e.g., high hydrophilicity and swelling properties) [1,2]. Figure 1 describes the characteristics of nanogel drug delivery systems related to their nanoparticle nature. In particular, the nanogels could enter and accumulate in tumor and inflammatory tissues due to their nanoscale size, known as the enhanced permeation and retention effect (EPR) (Figure 1a). The nanogel surface could be modified via stealth-coating (e.g., pegylation), which ensures avoidance of opsonins and prolongs circulation in the blood stream (Figure 1b). Huppertsberg et al. prepared PEGylated squaric ester-based nanogel and observed minor uptake from macrophages as well as absence of aggregation and increase in the size after incubation with human blood plasma, indicating lack of protein corona [3,4]. Additionally, their surface could be decorated by the attachment of targeting ligands, which further provides a great opportunity for selective drug delivery in certain cells that overexpress specific receptors (Figure 1c). For instance, polycarbonate nanogels were coated with CD4+-specific peptide in order to enhance the delivery of Toll-like receptor 7/8 agonist into immune cells [5]. The nanogels could protect the loaded active molecules against either in vitro (oxygen, light) or in vivo (enzymes, pH) harmful conditions (Figure 1d).

**Figure 2:**
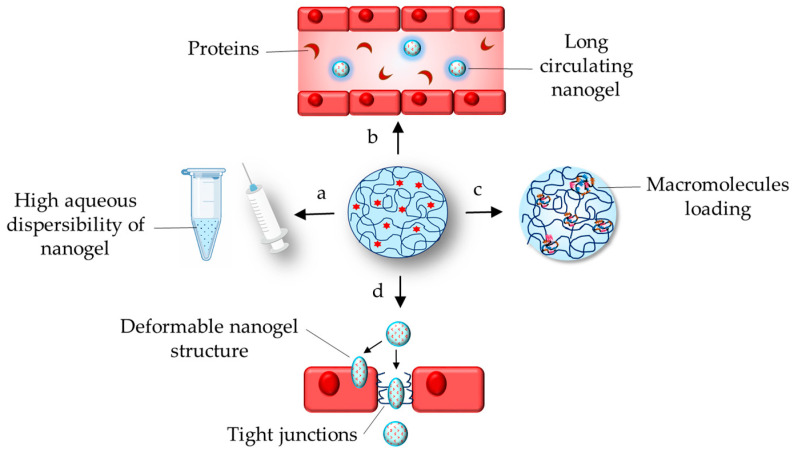
Schematic presentation of the specific advantages of the nanogels originating from their hydrogel structure: high aqueous dispersibility giving an opportunity for various routes of administration (**a**), reduced adsorption of proteins on the hydrophilic nanogel surface that results in a long circulation (**b**), effective loading of low and high molecular therapeutics (**c**), and improvement of trans/paracellular transport due to the soft and deformable nanogel structure (**d**).

The properties related to the hydrogel nature of nanogels are presented in Figure 2. In particular, the hydrophilicity and swelling ability determine high aqueous dispersibility and reduced adsorption of proteins in in vivo circulation (Figure 2a,b). The aqueous dispersibility allows their administration by various routes, including parenteral, whereas the reduced adsorption of proteins prolongs their circulation. In addition, the swelling ability of nanogels could resist platelet adhesion [6]. The nanogels provide a great capacity for loading different therapeutics independent of their molecular weight, solubility, and origin (synthetic or natural). Small active molecules, hydrophilic or hydrophobic, could be loaded by physical entrapment or chemical bonding to polymer groups. For instance, paclitaxel as well as interferon gamma (IFN-γ) were successfully loaded in hyaluronic acid nanogel, which resulted in enhanced anticancer effects on lung carcinoma A549 cells and alleviated toxic effects on healthy HEK293 cells [7]. On the other hand, the high porosity and cationic charge of some of the nanogels are crucial properties for effective loading of macromolecular therapeutics, including oligonucleotides and therapeutic proteins (Figure 2c). For example, synthetic long peptides were successfully loaded with a high efficiency (86.7–100%) in cationic dextran-based nanogel via electrostatic interactions [8]. As mentioned above, the nanogels would be able to protect these therapeutics against aggregation, denaturation, or enzymatic degradation. The loading of the enzyme paraoxonase-1 in cross-linked PEG-based nanogels maintained the catalytic activity of the enzyme after incubation in a medium mimicking intracellular glutathione concentration (10 mM) [9]. Numerous reviews have presented examples for the development of nanogels as platforms for the delivery of nucleic acids, peptides, and proteins, as well as imaging and contrast agents [10,11,12,13,14,15,16,17]. Furthermore, dual loading of therapeutics is an additional advantage of nanogels [18,19,20]. The simultaneous delivery of quercetin and paclitaxel loaded in folic acid–gelatin–Pluronic P123 nanogel resulted in enhanced antitumor effects on breast cancer MCF-7 and cervical cancer HeLa cells as well as in vivo in MCF-7 tumor-bearing mice [19]. Finally, the nanogels are soft and deformable, which are very important properties that could enable their transport through or between the cells (Figure 2d). For example, Sun et al. developed a nanogel possessing ultrasound-responsive deformation by cross-linking polylysine and Pluronic F127 with genipin [21]. The scientists observed the high ability of the nanogel to penetrate into the deep area of xenografted tumor under external ultrasonic stimulation due to the enhanced deformability of the formulation.

The nanogels can be prepared by two main methods, in particular physical or chemical cross-linking of polymer chains [22]. Physical crosslinking is achieved by self-assembly of amphiphilic or oppositely charged polymers, whereas chemical cross-linking is achieved by covalent linkages between different functional groups present on the polymer chains. The present review is not dedicated to the methods for the preparation of nanogels, taking into consideration the existence of previous reviews in this area [23,24].

The review is focused on the new achievements revealing the capacity of nanogels to overcome biological membranes. The biopharmaceutical behavior of nanogels is closely related to the characteristics of the polymeric carriers as well as the mechanism of drug loading. In this view, the properties of natural and synthetic polymer carriers are discussed with a focus on their ability for drug loading and membrane transport. The natural polymers are usually biocompatible and biodegradable. Furthermore, they could form nanogels under mild conditions (without surfactants, catalysts, etc.), particularly ionic gelation (mostly polysaccharides) or thermal denaturation and aggregation of proteins. On the other hand, synthetic polymers could be precisely synthesized (e.g., exact molecular weight, desired rate of degradation, etc.). As noted, drug loading and release processes are also discussed. Special attention is given to the different mechanisms, including stimuli responsiveness depending on internal biological stimuli. Finally, the challenges related to nanogel transport through the barriers presented in parenteral, oral, ocular, nasal, and dermal routes of administration are considered.

## 2. Nanogels Based on Natural Polymers

The use of natural polymers for the preparation of nanogels is gaining high interest currently owing to their advantages such as biodegradability, biocompatibility, functionality, the possibility for modifications, etc. The most used natural polymers for the last years are polysaccharides such as chitosan, dextran, hyaluronic acid, and pectin, as well as some proteins (Table 1).

### 2.1. Chitosan

Chitosan is a linear polysaccharide that is produced from chitin and consists of D-glucosamine and N-acetyl-D-glucosamine units, linked via β-(1→4) glycosidic bonds. The most important characteristics of chitosan are the degree of deacetylation, the pKa of its amino groups (6.5), and the molecular weight. Deacetylation degree is related to the number of amino groups, whereas pKa and molecular weight influence its solubility [40]. The presence of the amino groups determines its capacity for improvement of nanogel transport. In particular, the cationic properties in pH-neutral physiological fluids provide an opportunity for electrostatic interactions with the negatively charged membranes of the epithelial cells and the mucin in the mucus [41]. Rosental et al. reported that the protonated form of chitosan decreases both the transepithelial and paracellular resistance in an in vitro cell model that suggests its absorption-enhancing effect [42].

The amino groups in the molecule of chitosan could interact with negatively charged polymers, which is a prerequisite for its application as a nanogel carrier [43]. The hydrophilicity of chitosan could be modified by the attachment of hydrophobic units that bring amphiphilic properties to the polymer. Such amphiphilic polymers give an opportunity for self-assembly and more effective loading of hydrophobic drugs. For example, deoxycholic acid-modified glycol chitosan self-assembled in a nanogel that was capable of loading the hydrophobic antidiabetic drug palmitoyl acylated exendin-4 (Ex4-C16), providing slower drug release and longer hypoglycemic effects [44]. Essential oils of *Syzygium aromaticum* and *Cinnamomum ssp*. were successfully loaded into cinnamic acid-grafted chitosan nanogel [25]. The nanogel particles were characterized by high encapsulation efficiencies (74% and 89%, respectively) and small size (176.0 ± 54.3 nm and 263.0 ± 81.4, respectively). The authors observed enhanced antifungal effects of the loaded oils in the nanogel. Chitosan is known to be non-toxic, biodegradable, and biocompatible [45]. Thus, chitosan nanogels could be administered by different routes, including parenteral. Pereira et al. have examined glycol chitosan nanogels by hemolysis and whole blood clotting time assays and reported their blood compatibility [46]. Oral administration of nanoparticles containing chitosan and alginate in different ratios also proved the safety profile of such a biopolymeric nanosystem [47].

As noted, the amino groups of chitosan are a crucial factor for the development of nanogels. For example, nanogel particles were obtained by the formation of amide linkages between the amino groups of chitosan hydrochloride and the carboxyl group of carboxymethyl starch by applying carbodiimide cross-linking [48]. The resulting particles possessed a spherical shape and viscoelastic properties typical for gels. The nanogel ensured high loading of curcumin and pH-responsive delivery of the drug into simulated gastrointestinal medium. In another study, nanogels from chitosan and carboxymethyl chitosan have been obtained via electrostatic interactions between the amino groups of both polymers and the negatively charged carboxyl groups of carboxymethyl chitosan [49]. The obtained nanogel particles were characterized by a spherical shape and a mean diameter of 171 nm. They provided a high mucin-binding ability and pH-responsive release of the loaded antibacterial drug rifaximin. The authors concluded that the nanogel particles could be an effective mucoadhesive drug delivery system with a targeted release in the bacterial infection site. Nanogel particles were obtained by covalent cross-linking between the amino groups of chitosan and the carboxylic acid groups of benzoic acid through a carbodiimide reaction [50]. The nanoparticles were spherical, less than 100 nm, and rosemary essential oil was successfully loaded (80% encapsulation efficiency). The encapsulated rosemary oil showed an enhanced antibacterial activity against *Staphylococcus aureus* compared to the non-encapsulated oil. Essential oils from *Mentha piperita* were also incorporated into chitosan nanogel cross-linked with tripolyphosphate [51]. The formulation showed improved biofilm inhibition against *Streptococcus mutans* through downregulation of genes related to biofilm formulation and virulence. Thus, the developed nanogel was considered as a potential antibiofilm agent in toothpaste or mouthwashing formulations. The minimum inhibitory concentration of thymol for *Staphylococcus aureus*, *Acinetobacter baumanii*, and *Pseudomonas aeruginosa* strains decreased 4 to 6 times and the expression of biofilm-related genes was significantly down-regulated when it was loaded into chitosan nanogel [26]. The system possessed an average size of 82.71 ± 9.6 nm, an encapsulation efficiency of 76.54 ± 0.62%, and stability up to 60 days at 4 °C. Moreover, the authors observed negligible toxicity in healthy human embryonic kidney cells (HEK293). Glycol chitosan and aldehyde-capped poly(ethylene oxide) were used for the preparation of hollow nanogel particles that were loaded with the protein urokinase-type plasminogen activator [52]. The nanogel particles were sensitive to diagnostic ultrasound, and the sonothrombolysis performance of the urokinase-loaded particles was tested in vivo on an acute ischemic stroke rat model. The nanogel showed enhanced thrombolysis efficiency of the protein and prolonged blood circulation. The wound healing capacity of trinitroglycerin was enhanced after its encapsulation in chitosan nanogel, which was characterized with 70.2% encapsulation efficiency and approx. 96 nm mean size [27]. The authors observed enhanced migration of human umbilical vein endothelial cells (HUVECs) and lack of cytotoxicity. Moreover, there was complete wound closure, skin component formation, promoted collagen deposition, and decreased scar width after topical application of the nanogel on rat wounds. It was found that the accelerated wound healing was due to promotion of collagen deposition, angiogenic activity, and decrease in inflammation.

### 2.2. Dextran

Dextran is a polysaccharide that is produced from *Leuconostoc mesenteroides* and some other bacteria strains. It is appropriate as a nanogel carrier because of good biocompatibility, biodegradability, and the presence of a large number of chemically reactive hydroxyl groups. The latter allows different modifications of dextran and the formation of various structures, including three-dimensional nanogels. Furthermore, the presence of functional groups of dextran in the nanogel shell could be used for surface decoration and improvement of interactions with cell membranes. For instance, a dexamethasone-loaded nanogel composed of dextran and lysozyme was developed via a Maillard reaction followed by heat gelation [53]. The nanoparticles were further conjugated with an antibody directed to intercellular adhesion molecule-1 (ICAM), aiming to target pulmonary endothelium and to alleviate acute pulmonary inflammation. Nanogels from polyaldehyde dextran and cystamine have been prepared via disulfide-containing Schiff base formation applying the microemulsion method [54]. Doxorubicin was loaded by covalent conjugation on the chains of polyaldehyde dextran via Schiff base linkages. The formulation showed dual pH and redox stimuli responsiveness. Lower pH led to hydrolysis of the Schiff bases, whereas the high concentration of glutathione provoked breaking of the disulfide bonds. Sun et al. prepared tocopheryl polyethylene glycol succinate (TPGS)—grafted dextran nanogel particles cross-linked by ortho ester-based compound [55]. The nanogel particles were loaded with doxorubicin (194 nm, 83% encapsulation efficiency) and showed pH-dependent release being faster at a pH of 5.5. In vitro cell cytotoxicity and efflux capacity studies proved the ability of the TPGS-grafted nanogels to inhibit the P-glycoprotein efflux pump and to enhance the antitumor effect of doxorubicin in multidrug-resistant breast cancer cells. Nanogels from soybean protein isolate and dextran have been obtained via Maillard reaction due to the aldehyde groups of dextran and protein self-assembly [56]. The nanogel particles were spherical, had a core–shell structure, a diameter of 104 nm, and high stability at different temperatures and ionic strengths. Curcumin was successfully loaded into soybean–dextran conjugate nanogels, achieving approximately 89% encapsulation efficiency [30]. The loading resulted in enhanced radical-scavenging ability and improved photo- and thermal stability of curcumin. Nanogels for nasal vaccine delivery have been obtained through electrostatic interactions between dextran sulfate (anionic sulfate groups) and ε-polylysine [28]. Ovalbumin loading significantly changed the size of the nanogel particles. In particular, at 40.5% encapsulation efficiency (1 h incubation during loading), the size was 214 nm, whereas at 95% encapsulation efficiency (24 h incubation), it increased to 624 nm. This diameter, the zeta potential (30.5 mV), and the sustained release (43% for 72 h) were considered appropriate characteristics for the local nasal delivery. Ovalbumin was also successfully loaded into another nanogel based on l-arginine-modified dextran. Controlled release, enhanced uptake, storage, and immunogenic maturation in dendritic cells, as well as M1 polarization of macrophages in vitro, delayed clearance, and finally stronger inducement of cellular and humoral immunity in vivo were achieved [29].

On the other hand, the dextran nanogels could provide better biocompatibility of magnetic nanoparticles. For instance, iron oxide nanocrystals were inserted in dextran nanogel, aiming to achieve a magnetic resonance imaging [57]. The nanogel system combined the superparamagnetic properties and biocompatibility of dextran.

### 2.3. Hyaluronic Acid

Hyaluronic acid is a linear polysaccharide, an anionic glycosaminoglycan, composed of disaccharide units of D-glucuronic acid and N-acetyl-D-glucosamine. The polymer is biodegradable, biocompatible, and non-toxic. The existence of carboxylic and hydroxyl acid groups provides a great possibility for interaction with cationic polymers or drugs as well as further functionalization and development of targeted nanogels. Another advantage of nanogels based on hyaluronic acid is that the release of the incorporated drugs could be controlled by the enzyme hyaluronidase, which is responsible for the biodegradation process. Furthermore, many cancer cells overexpress hyaluronic acid-specific binding receptors that give an opportunity for active targeting of hyaluronic acid-based nanogels loaded with anticancer drugs. For instance, Zhang et al. prepared PEGylated hyaluronic acid nanogels and achieved hypoxia-stimulated and hyaluronidase-triggered interleukin-12 release for cancer treatment [58]. Biotin-functionalized, cholesterol-grafted methacrylated hyaluronic acid nanogel has been loaded with paclitaxel through hydrophobic interactions [31]. The formulation showed a spherical shape, a mean diameter of 149 nm, and an encapsulation efficiency of approximately 91%. Biotin ligands provided biotin receptor-mediated cell uptake in mouse breast cancer 4T1 cells and an increased tumor accumulation in 4T1 tumor-bearing mice. The antiangiogenic drug vatalanib and the near-infrared photothermal agent indocyanine green were simultaneously encapsulated into a nanogel based on histidine-modified methacrylated hyaluronic acid, which was further included into exosomes [33]. Targeted delivery, resulting in inhibited tumor angiogenesis and enhanced antitumor effect in vitro and in vivo, was observed. A recent study compared the characteristics of nanogels and PLGA-nanoparticles as pulmonary systems for azithromycin delivery [59]. The nanogel particles were formulated from octenyl succinic anhydride-modified hyaluronic acid. The formulation showed a small mean diameter (159 nm), a negative charge (−17 mV), and an encapsulation efficiency of 45%. Interestingly, the nanogel particles presented weaker interactions with mucin and consequently more appropriate mucus-penetrating properties than PLGA nanoparticles (92 nm, −27 mV). This phenomenon suggested that the nanogels could reach more efficiently the bacterial biofilm, overcoming the surrounding mucus layer. Both formulations penetrated into biofilms of *Pseudomonas aeruginosa* and allowed removal of the preformed biofilms at lower doses compared to the non-encapsulated azithromycin.

As it was mentioned above, in some cases the hydrophilicity of the biopolymers could be reduced, aiming to improve the loading of hydrophobic drugs. An alkyl glyceryl hyaluronic acid, a hydrophobized hyaluronic acid, was used for the preparation of nanogels by the dialysis method [32]. The nanogel particles were loaded with a hydrophobic drug, amphotericin B, and their characteristics regarding ocular administration were examined. The authors discovered that the formulation interacted with mucin, which could lead to extended contact time with the cornea, lowered toxicity of the drug, and demonstrated high fungal selectivity. It was suggested that the latter was due to interaction between the hyaluronic acid and the chitin in the fungal cell. In another study, Zoratto et al. developed a cholesterol-grafted hyaluronic acid copolymer that forms a nanogel with a hydrophobic cavity of cholesterol units and a hydrophilic surface of hyaluronic acid [60]. The formulation was appropriate for loading hydrophilic (tobramycin and diclofenac sodium salt) as well as hydrophobic drugs (dexamethasone and piroxicam). The nanogel particles were suitable for topical ocular delivery since they combined the mucoadhesive properties of hyaluronic acid and the capacity of cholesterol to penetrate through corneal epithelial cells. Cholesteryl-group-bearing pullulan has been loaded with a non-toxic subunit fragment of *Clostridium botulinum* type-A neurotoxin BoHc/A for intranasal vaccine delivery [61]. The drug-loaded formulation was retained by the nasal mucosa for more than two days and induced strong tetanus-toxoid-specific systemic and mucosal immune responses. Also, the vaccine did not accumulate in the olfactory bulbs and brain, which confirmed its safety.

### 2.4. Pectin

Pectin is a linear polysaccharide that consists of (1-4)-linked D-polygalacturonic acid residues. Like the other polysaccharides, pectin is biodegradable, biocompatible, and nontoxic. The carboxyl groups provide the anionic properties of the biopolymer that determine a well-pronounced capacity for nanogel formulation via electrostatic interactions. Biodegradable nanogel particles were prepared by self-assembly of pectin and lysozyme (positively charged protein) through electrostatic interaction and consequent gelation of lysozyme by heat treatment [62]. The resulting nanogel particles were spherical (average diameter of 109 nm) and stable in a pH range between 6.0 and 11.0. The narrow size distribution did not change after storage for 90 days at 4 °C. Another study reported the construction of nanogels via hydrophobic and electrostatic interactions between low-density lipoprotein from egg yolk and pectin [63]. The pH- and heat-induced assembly resulted in spherical and smooth nanogel particles with a mean diameter of 58 nm and a negative charge (−41 mV). An innovative nanospray drying technology was optimized to obtain nanosized powder of nanogels with excellent dispersibility in water. Nanogel from pectin and alginate was prepared via the ion gelation method [34]. Naringin-hydroxypropyl-β-cyclodextrin complex was loaded in the nanogel, achieving approximately 79% encapsulation efficiency.

### 2.5. Proteins

Albumin is a globular protein that possesses pronounced aqueous solubility, biocompatibility, and biodegradability. Albumin possesses functional groups in its structure, namely carboxylic and amino groups, which at certain pH values could participate in electrostatic interactions with oppositely charged polymers or drugs [64]. Thermally induced denaturation and aggregation of albumin could contribute to the formation of a three-dimensional gel structure. Furthermore, the presence of albumin on the nanoparticle surface could repulse other proteins and inhibit the formation of an unwanted protein corona. The latter is related to the possibility to prolong the blood circulation of the formulations by reducing the macrophage phagocytosis [65]. All these properties of albumin make it an appropriate polymer for the preparation of nanogels. Bashiri et al. prepared nanogels from bovine serum albumin and gum arabic aldehyde via electrostatic interactions between the amino groups of albumin and aldehyde groups of the gum arabic (Schiff base interactions) under mild conditions [35]. The resulting nanoparticles contained 2.37% 5-fluorouracil and showed a mean diameter of 231 nm. The formulation demonstrated good hemocompatibility and appropriate parenteral administration. Liu et al. prepared nanogels from carboxymethyl cellulose and bovine serum albumin for simultaneous delivery of radionuclide ^131^I and camptothecin for combined chemo-radioisotope therapy of cancer [66]. The nanogel particles were produced at a specific pH of the aqueous mixture of both polymers (pH was adjusted to the pI of BSA (5.0)) and consequent heating for 60 min at 70 °C. The nanoparticles were spherical in shape with an average size of about 120 nm, hemocompatibility, and prolonged blood circulation. In vivo experiments on tumor-bearing C57BL/6 mice proved that the combined therapy significantly improved the antitumor effect in comparison with each therapy separately. Curcumin-loaded nanogels comprising an albumin core and a folic acid-functionalized amylopectin shell were developed via thermal gelation [67]. The nanogel particles had small diameters (90 nm) and negative zeta potentials of −24 mV. The shell of the nanogel particles hindered the digestion in gastrointestinal medium because of B- and V-type liquid crystalline polymorphism, resisted the action of amylases, protected the protein core from proteolytic enzymes, and decreased the degradation of curcumin. In vitro tests on human colon adenocarcinoma HT29 cells, which are overexpressing folate receptors, showed enhanced internalization, retention, and improved anticancer effect of the drug-loaded nanoparticles. Highly stable nanogels (in the pH range from 2 to 10.5) based on ovalbumin and chitosan were obtained by pH- and temperature-induced gelation [68]. It has been reported that a part of chitosan chains was entrapped in the nanogel core during the heating, while another part of chitosan chains formed the shell. Recently, bovine serum albumin and chitosan were applied for the preparation of nanogel as a carrier of the anticancer drug doxorubicin [36]. The nanogel was obtained by electrostatic gelation (at pH = 4.54) and consequent heating (at 78 °C). The nanogel system (diameter of 30 nm) protected doxorubicin from light degradation and enhanced the anticancer effect of the drug on epidermoid squamous skin carcinoma A-431 cells. Nanogel particles were obtained from albumin and pullulan through a Maillard reaction combined with heat treatment [37]. The curcumin-loaded nanogel particles possessed a diameter less than 200 nm and appropriate storage stability for 30 days.

Lysozyme is a globular protein (14.3 kDa) extracted from egg whites. The most important prerequisites for its wide use as a nanogel carrier are its biocompatibility, biodegradability, and positive charge. Lysozyme solution is positively charged in medium with a pH lower than 9.4, which is lower than its isoelectric point (pI = 10.7). On the other hand, it provides plasticity, elasticity, and water-binding capacity for gelled structures. Typically, lysozyme is investigated as a carrier of nanogels prepared by electrostatic assembly with polysaccharides. Nanogel particles composed of lysozyme and carboxymethylcellulose were prepared by self-assembly and consequent heat treatment, leading to the appearance of electrostatic and hydrophobic interactions [69]. The denaturation of the lysozyme under heating enabled intermolecular hydrophobic interactions that resulted in a tight and stable nanogel structure. Importantly, the stable structure protected the encapsulated tea polyphenols against pH- and heat-induced degradation. Carboxymethyl chitosan–lysozyme nanogels were loaded with tea polyphenols, silver nitrate, or chlorhexidine by Zhou et al. [39]. All three systems possessed mean diameters below 200 nm, spherical shape, negative zeta potential, and enhanced antibacterial effect against *Streptococcus mutans*. Nanogel particles consisting of a lysozyme core and dextran shell were developed in two steps: (1) formation of lysozyme−dextran conjugates through the Maillard reaction and (2) heating of conjugate solution above the denaturation temperature of lysozyme [70]. The resulting nanogel particles were spherical in shape, possessed a hydrodynamic diameter of 200 nm, and had high stability independent of changes in pH and ionic strength. The same method was applied for the preparation of 5-fluorouracil-loaded nanogel that consisted of lysozyme and sodium carboxymethyl cellulose [71]. In this case, the drug also participated in the formation of intermolecular complexes by hydrogen bonding between its amide groups and the carboxyl groups of sodium carboxymethyl cellulose.

Mucoadhesive cationic disulfide-cross-linked poly(l-lysine)–poly(l-phenylalanine-co-l-cystine) nanogel particles were loaded with 10-hydroxycamptothecin for treatment of bladder cancer [72]. The formulation showed high intracellular concentration in cancer bladder 5637 cells due to the interference between the positive charge of the formulation and the negatively charged cell membranes. On the other hand, the high glutathione concentration in the cells triggered the release of the active substance via breaking of the disulfide bonds. Glutathione-responsive methylated poly(ethylene glycol)-poly phenylalanine)-poly(cystine) block copolymer was used for the development of a shikonin-loaded nanogel (63 nm) [38]. The study group observed redox-responsive release of shikonin, biodistribution in the tumor site, and enhanced antitumor effects. Feng et al. prepared disulfide-cross-linked methoxy poly(ethylene glycol)–poly(L-phenylalanine-co-L-cystine) nanogels and loaded methotrexate for treating rheumatoid arthritis [73]. The nanoparticles provided targeted delivery in inflamed tissues due to the high presence of glutathione and breaking of the disulfide bonds. Better biodistribution and antitumor effect of methotrexate in mice was observed when it was loaded into the nanogel particles. Mannose-conjugated antimicrobial polypeptide poly(arginine-r-valine)-mannose was cross-linked with Zn^2+^ for the formulation of nanogels [74]. The cationic arginine in the formulation was responsible for the improved antibacterial effect against *Staphylococcus aureus* due to the enhanced electrostatic interactions with the phosphate groups of the bacterial membrane. The optimized nanogels representing the mannose residues on their surface showed a more selective activity and hemocompatibility.

## 3. Nanogels Based on Synthetic Polymers

The synthetic polymers are of interest for preparing nanogels owing to the opportunity for synthesizing polymers with desirable characteristics and reproducibility, which results in the preparation of nanoparticles with exact properties. The most common synthetic polymers used for the formulation of nanogels currently are poly(acrylic) acid, poly(methacrylic acid), polyethylenimine, and their derivatives (Table 2).

### 3.1. Poly(acrylic acid)

Poly(acrylic acid) is a polymer containing monomers of acrylic acid, which is negatively charged at certain pH (pKa = 4.5) and could absorb large amounts of water. These characteristics make it significantly appropriate for preparing gel networks, due to the opportunity for participating in electrostatic interactions with oppositely charged polymers, cross-linkers, or active substances. On the other hand, the pH-dependent protonation of the carboxylic groups is a prerequisite for obtaining pH-sensitive drug release. Mickiewicz et al. prepared poly(acrylic acid) nanogels cross-linked with N,N′-methylenebisacrylamide via precipitation polymerization reaction in water [84]. The authors observed significant changes in size and zeta potential as well as very high swelling coefficient of approximately 4000 when the pH increased from 2 to 8. Biodegradable and pH-responsive nanogels from Pluronic F127 and poly(acrylic acid) were prepared due to interactions between the ether functional groups of Pluronic F127 and the carboxylic groups of the acid [75]. The loading of terbinafine hydrochloride increased proportionally to the swelling and porosity of the nanogels. Due to pH-dependent swelling and release, the obtained nanogels showed better in vitro and in vivo antifungal activity against *Candida albicans* than the commercial product (Lamisil 1%). Skin irritancy tests showed that the drug-loaded nanogel particles do not cause erythema and edema and are safe. Poly(acrylic acid) and polyvinylpyrrolidone have been used for the preparation of nanogels for ocular administration via the ionizing radiation method [85]. Hydrogen bonds between the two polymers have been formed. The formulation showed mucoadhesive properties, which contributed to an enhanced effect on the dry eye model in comparison with the commercial Vidisic gel 0.2%. Zhu et al. developed a nanogel from poly(acrylic acid) with loaded mitoxantrone and metallic manganese ions. It was characterized by a mean diameter of 110 nm and spherical shape, negative zeta potential, and encapsulation efficiency of 77.9% for mitoxantrone and 18.2% for manganese ions. The authors observed pH-dependent release, more pronounced in slightly acidic medium, corresponding to lysosomal pH, as well as enhanced anticancer effect in vitro in colon carcinoma CT29 cells and in vivo in CT26 tumor-bearing mice [76]. Nanogels from poly(acrylic acid) with different molecular weights were conjugated with bombesin peptide and radiolabeled. Increased internalization in prostatic adenocarcinoma PC-3 cells was observed [77].

### 3.2. Poly(methacrylic acid) and Its Derivatives

Poly(methacrylic acid) and its derivatives are built from methacrylic acid monomers and possess free carboxylic groups. The pKa of the polymer is 4.8, and in neutral conditions it is negatively charged. Similarly to poly(acrylic acid), it has the ability to absorb water, and owing to its functional groups, it can form electrostatic bonds with cationic polymers or drugs, resulting in nanogel formation. Also, this polymer could provide pH-responsive drug delivery. For instance, Salehi et al. developed dual-stimuli responsive nanogels based on poly(N-isopropylacrylamide-dimethylaminoethyl methacrylatequaternary ammonium alkyl halide-methacrylic acid) and poly(N-isopropylacrylamide-dimethylami-noethyl methacrylate quaternary ammonium alkyl halide-meth-acrylic acid-hydroxyethyl methacrylate) [86]. The nanogel particles delivered doxorubicin and methotrexate depending on temperature (more rapid at 40 °C than at 37 °C) and pH values (faster at pH 4–5.5, but arrested at pH 7.4). The antioxidant edaravone was loaded into glutathione-conjugated poly(methacrylic acid) nanogel with almost 100% encapsulation efficiency for drug delivery in the brain [78]. The nanogel particles showed a hydrodynamic diameter of 199 nm and a negative zeta potential (−25 mV). The simultaneous delivery of the antioxidant and glutathione elevated spatial memory in Wistar rats. Amoxicillin was encapsulated into poly(methacrylic acid) nanogel particles cross-linked with ethylene glycol dimethacrylate [87]. The nanogels were prepared via thermally initiated precipitation polymerization, and the size depended on the concentration of the cross-linker. At 67% concentration, the size was less than 300 nm, whereas at 33% cross-linker, the size was 480 nm. The authors observed controlled sustained release of the drug without a burst effect, improved stability of the amoxicillin in acidic and basic conditions, and improved antibacterial activity against Staphylococcus aureus and Escherichia coli. Such poly(methacrylic acid) nanogel cross-linked with ethylene glycol dimethacrylate (diameter less than 600 nm) was also prepared for oral delivery of metronidazole [88]. Sustained release with no burst effect was observed, with approximately 12% at pH 1.2 (for 4 h), 33% at pH 6.8 (for 6 h), and 60% at pH 7.2 (for 50 h). These results proved that the formulation is appropriate for oral application and drug delivery into the colon, overcoming the side effects in the stomach. The encapsulation-stabilized metronidazole provided enhanced long-term antibacterial activity against Bacteroides fragilis and improved safety in mouse L929 fibroblasts. Biocompatible and non-toxic poly(ethylene glycol)-poly(methacrylic acid) nanogel was successfully loaded with olmesartan, achieving more than 80% encapsulation efficiency and a mean size of 300 nm [79]. The authors observed increased solubility of the drug, particularly 12.3-fold in a buffer with pH = 1.2, 13.29-fold in a buffer with pH = 6.8, and 10.2-fold in water. Moreover, there was a pH-dependent release, more pronounced in the medium with pH = 6.8.

The nanogels based on poly(methacrylic acid) could incorporate small metal nanoparticles intended for photothermal therapy. For example, pegylated nanogels designed with poly(2-[N,N-diethylamino]ethyl methacrylate) (PEAMA) cores containing gold nanoparticles were prepared through the reduction in Au(III) ions with the participation of the tertiary amino groups of PEAMA [89]. The authors reported that the nanogel was a selective and noninvasive system for cancer photothermal therapy, particularly that the nanogel was not cytotoxic in the absence of irradiation and cytotoxic under irradiation.

### 3.3. Polyethylenimine

Polyethylenimine (PEI) is a polymer, consisting of monomers with two methylene groups and terminal amino groups. It could be linear (semi-crystalline) or branched (amorphous). It is an ideal carrier or cross-linking agent for the preparation of nanogels because of the presence of cationic groups and the branched structure. It also could possess antibacterial effects since the cationic amino groups may interact with positively charged components of the bacterial cell wall. One of the first synthetic nanogels was developed by cross-linking branched PEI with an amino-reactive PEG linker, applying the emulsification–solvent evaporation method [90]. After that, different studies examined various nanogels of PEI. Since polyethylenimine possesses efficient transfection ability, heparin–polyethylenimine cationic nanogel particles have been prepared for gene delivery for lung cancer therapy [91]. A plasmid with high expression efficiency of interleukin-15 has been conjugated to the nanogels that were i.v. applied in mice. The encapsulation of the plasmid resulted in its high distribution level in the lung and a lower tumor metastasis index compared with other treatment groups. Polyethylenimine nanogels have been loaded with cancer cell membrane-camouflaged iron oxide nanoparticles for dual delivery of the anticancer drug docetaxel and CD47 siRNA [81]. This formulation showed targeted redox-responsive delivery in the tumor microenvironment and could be used in magnetic resonance imaging and treatment of tumors by a combination of chemotherapy and gene silencing. In another study, poly(ethylenimine)-poly(ethylene glycol) nanogels were loaded with transforming growth factor-β1 siRNA and ultrasmall iron oxide nanoparticles, aiming for gene therapy and T1-weighted magnetic resonance (MR) imaging. The authors observed enhanced MR imaging performance, enabled internalization into the cytoplasm of sarcoma cancer S180 cells, and inhibition of the growth of a subcutaneous sarcoma tumor and lung metastasis in a mouse model in vivo [83]. Polyethyleneimine-coated iron oxide nanoparticles were loaded in a nanogel based on γ-polyglutamic acid [92]. The iron oxide nanoparticles were encapsulated into the nanogel for application as a contrast agent for MR imaging. The size of the nanogel particles was 152 nm, and they were reported as water-dispersible, colloidally stable, and non-cytotoxic in a certain concentration range. Hydroxyapatite nanoparticles loaded with doxorubicin were inserted in Pluronic P123-branched polyethylenimine nanogel. The presence of amino groups in the structure of PEI could contribute to enhanced cellular uptake as well as a pH-dependent release, and this resulted in enhanced anticancer effects in liver cancer HepG2 cells [82]. Gold nanoparticles and gadolinium have been loaded into polyethylene glycol-modified polyethylenimine via chelation [93]. After that, this formulation was used as a cross-linker of alginate in order to obtain nanogel. The loaded nanogel particles were characterized with small diameter (83 nm), colloidal stability, safety, and MR/CT (magnetic resonance/computed tomography) imaging ability.

## 4. Mixed Nanogels

Both natural and synthetic polymers possess cationic or anionic functional groups, which allow their interaction and formation of nanogels. Such mixed nanoformulations are characterized by the advantages of both types of polymers and could fulfill the desired properties depending on the application (Table 3).

Mixed nanogels from hyaluronic acid and polyethylenimine (diameter less than 300 nm) were obtained for anticancer treatment [94]. The authors observed sustained release of doxorubicin for 15 days and improved internalization of the nanoparticles in vitro because a hyaluronan receptor was involved in the nanogel internalization. Covalently cross-linked hybrid nanogels composed of chitosan and poly(methacrylic acid) were characterized with excellent structural stability and regulation of the release process depending on the typical pH range of 5–7.4 found in pathological tissues [98]. For comparison, the nanogels formed by non-covalent physical association showed a significant change in the structure and composition upon exposure to the same pH values.

Poly(acrylic acid)/deoxycholic acid-modified chitosan nanogel was obtained for encapsulation of insulin through van der Waals, electrostatic, hydrogen interactions, and CH/π stacking [95]. The authors observed spherical shape of the nanoparticles with mean diameter of 209 nm, low polydispersity index and zeta potential of −27.5 mV. Importantly, the nanogel preserved the structure of the protein. Pullulan and poly(acrylic acid) were used for the preparation of a nanogel that was modified with bovine serum albumin and folic acid [96]. Doxorubicin was successfully loaded, and the release pattern showed more pronounced release in acidic medium. In vitro tests revealed an enhanced cytotoxic effect of doxorubicin on MCF-7 cells, decreased adverse effects in healthy L929 fibroblasts, and improved cell internalization due to the folic acid. Nanogel from pectin and poly(acrylic acid) was prepared as a delivery system of rutin, achieving 86% drug content [97]. The nanogel provided pH-dependent release of rutin and enhanced anticancer effects on HepG2 (hepatocellular carcinoma), A549 (lung cancer), MCF-7 (breast cancer), and HCT-116 (colon cancer) cells. The antifungal drug luliconazole has been loaded into chemically cross-linked nanogels prepared from poly(acrylic acid) grafted sodium carboxymethyl cellulose [99]. The authors observed pH-dependent drug release corresponding to zero-order kinetics, enhanced permeation in comparison with the commercial product lulixol ex vivo, and improved antifungal effect against *Candida albicans*.

## 5. Drug Loading

**Figure 3:**
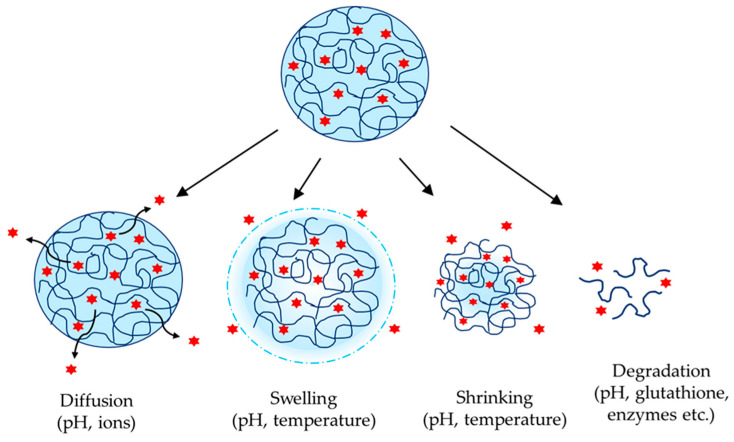
Schematic presentation of the different mechanisms of release processes.

Nanogels are able to incorporate active substances into their structure through physical entrapment or covalent bonding. Physical incorporation occurs mainly through electrostatic, van der Waals, hydrophobic, or hydrogen bonds. Covalent bonding occurs when both the active substance and the nanogel carrier possess appropriate functional groups. Both methods have some disadvantages, e.g., physical entrapment usually results in a burst release of the loaded drug, whereas chemical coupling could affect the drug’s activity. Both types of loading could appear with hydrophilic as well as hydrophobic drug molecules. On the other hand, the way of drug loading (physical or chemical approach), the characteristics of the drug and the carrier, the cross-linking density, and the introduction of stimuli-sensitive bonds are among the factors that influence the drug release process (Figure 3). As seen, all possible processes could depend on the pH value of the medium. In the case of diffusion, if the drug or polymer carrier has pH-dependent solubility, this definitely would influence the release process. Swelling and shrinking are closely related to the presence of polymer groups with pH-dependent protonation/deprotonation. The degradation process could be provoked by the insertion of pH-responsive bonds (acetal, ketal bonds, etc.) that would be disrupted at certain physiological pH value, e.g., at the acidic pH in the tumor interstitium and cellular lysosomes. Other types of bonds could also induce release through nanogel degradation, e.g., disulfide and diselenide bonds could be broken at high glutathione concentration (redox-responsive nanogels). In many cases, more than one of the listed factors could govern the release process, e.g., nanogels could be dual-responsive (pH- and thermo-responsive, pH- and glutathione-responsive bonds, etc.). For example, dual pH- and temperature-responsive nanogel might provide tumor-specific delivery of the drug, having in consideration the acidic pH and the local hyperthermia in tumor tissue [100]. To illustrate the described processes, some examples for loading and release of both drug categories (hydrophilic and hydrophobic) are discussed in the next sections.

### 5.1. Incorporation of Hydrophilic Compounds

The main characteristic of nanogel drug delivery systems is their hydrophilicity. Thus, the incorporation of hydrophilic substances would be easily accomplished, and different examples are listed in Table 4. Drug loading typically occurs through hydrogen bonds, van der Waals, hydrophobic, and electrostatic interactions between the drug molecule and the polymeric groups. Electrostatic interactions are very common because the presence of ionic groups in the drug molecules ensures their interaction with oppositely charged groups of the nanogel carrier. For example, doxorubicin was loaded into nanogels obtained from two naturally based products, in particular citric acid and pentane-1,2,5-triol [101]. The high encapsulation efficiency (95%) was explained with the electrostatic interactions between the carboxylic groups of citric acid and amino groups of doxorubicin. The drug release was faster in an acid medium (pH 5.0) compared to a slightly alkaline medium (pH 7.4) due to protonation of the amino groups of doxorubicin and the carboxyl groups of the nanogel carrier at pH 5.0. The protonation made the interaction weaker and accelerated the drug release in the acid medium. Similarly, doxorubicin loaded in alginate-poly[(2-dimethylamino) ethyl methacrylate] nanogel exhibited faster release in acid medium because of electrostatic repulsion due to protonation of doxorubicin and carboxyl groups of alginates [102]. Ma et al. simultaneously encapsulated doxorubicin and cisplatin into a nanogel based on hyaluronic acid [18]. Cisplatin was loaded via coordination interaction between the drug and the carboxyl groups of hyaluronic acid, whereas doxorubicin was loaded via electrostatic interactions. The nanogel released the drugs when the environmental pH value decreased (e.g., in a tumor tissue) because the coordination and electrostatic interactions between hyaluronic acid and both drugs became very weak. Nanogels composed of monomethoxy poly(ethylene glycol) conjugated to hyaluronic acid were loaded with the cationic protein cytochrome C through multiphysical interactions [103]. The nanogel particles were cross-linked by applying hypoxia-responsive cross-linkers that ensured the release of the protein in hypoxic tumor tissue. A cationic antibacterial peptide nisin was loaded in a chondroitin sulfate-based nanogel via electrostatic interaction [104]. The release of nisin was pH- and enzyme-responsive because of the presence of sensitive bonds in chondroitin sulfate. In particular, the nanogel particles degraded and released nisin under the activity of hyaluronidase secreted by Staphylococcus aureus.

Negatively charged oligonucleotides have been easily loaded in a nanogel composed of PEG and polyethylenimine (PEI) due to the cationic character of PEI [105]. It is important to note that the nanogel protected the loaded oligonucleotides from enzymatic degradation in mouse blood serum. In another study, amino-functionalization of cholesterol–pullulan nanogel resulted in cationic properties that further increased the loading of a model protein (bovine serum albumin) [106]. In fact, non-ionic cholesterol–pullulan nanogel particles also bound albumin due to hydrophobic interactions between the nanogel cholesterol groups and the hydrophobic domain of the protein. However, the amino-functionalized nanogels showed higher binding constants because, in this case, hydrophobic and additional electrostatic interactions between the cationic groups of pullulan and anionic albumin occurred. In in vitro conditions, the protein was released in the presence of an excessive amount of other proteins without disintegration of the nanogel structure. For example, chymotrypsin entrapped in the same nanogel formulation was released in the presence of albumin, probably because of the more hydrophobic properties (exchange mechanism) [107]. After intracellular uptake of the nanogels, the protein could be released by both processes, particularly (1) protein exchange and (2) disintegration of the nanogel structure by enzyme degradation of the pullulan [106].

One possible limitation of nanogels could be related to the difficult achievement of sustained release of hydrophilic drugs. The contribution of more than one type of interaction was considered as a possible approach to overcome the fast release. Doxorubicin was loaded in chitosan–alginate nanoparticles, and the authors reported the contribution of electrostatic, hydrophobic, and dipole–dipole interactions between the drug and sodium alginate [108]. The release test revealed the achievement of sustained release, particularly 91% and 68% released drug for 240 h in buffers with pH 5.5 and 7.4, respectively. The hydrophobic and dipole–dipole interactions as well as the stronger electrostatic attractions between doxorubicin and sodium alginate at pH 7.4 (both the amino groups of doxorubicin and the carboxyl groups of sodium alginate are ionized) ensured the slower release at pH 7.4. This pattern was considered advantageous since it would reduce the drug loss until it reaches the target tumor tissue. In another study, Kim et al. prepared nanogel particles (70 nm) from PEG-b-poly(L-glutamic acid) copolymer that were hydrophobically modified with L-phenylalanine methyl ester moieties [109]. The loaded doxorubicin was released from the hydrophobically modified nanogels in a slower manner (20% for 8 h) compared to the released amount from non-modified nanogels (85% for 8 h).

**Table 4:** Nanogels loaded with hydrophilic substances.

Composition of Nanogels	Active Substance	Mechanism of Release	Encapsulation Efficiency (%)	Reference
Zwitterionic poly(phosphorylcholine) and crosslinker composed of azobenzene bonds	Doxorubicin hydrochloride	Hypoxia-responsive degradation of nanogels	25.6	[110]
Alginate	Cisplatin	Degradation	37.8	[111]
Citric acid and pentane-1,2,5-triol	Doxorubicin hydrochloride	Diffusion through a porous matrix	95	[101]
Hyaluronic acid	Doxorubicin hydrochloride and cisplatin	pH-responsive	49.5 (doxorubicin) 29.6 (cisplatin)	[18]
Phenylboronic acid-modified hyaluronic acid and bis(methacryloyl) cystamine	Doxorubicin	pH- and glutathione-responsive destruction	83.3	[112]
Poly(2-methacryloyloxyethyl phosphorylcholine) crosslinked with diselenide bonds	Doxorubicin hydrochloride	Glutathione- and ROS-sensitive degradation	15.2	[113]
Chitosan	Bleomycin	pH-dependent swelling	55	[114]
Carboxymethylcellulose and lysozyme	Tea polyphenols	Diffusion	89	[69]
Sodium carboxymethyl cellulose and lysozyme	5-fluorouracil	pH-dependent swelling and erosion	30.5	[71]
Sodium alginate and poly(2-acrylamido-2-methylpropanesulfonic acid)	Caffeine	Diffusion and pH-dependent swelling	n.d.	[115]

### 5.2. Incorporation of Hydrophobic Compounds

Nanogels possess strongly expressed hydrophilicity, however, the variety of loading mechanisms provides an opportunity for encapsulation of hydrophobic drugs (Table 5). Many natural substances (essential oils, phenolic compounds, carotenoids, etc.) with remarkable pharmacological activities are highly hydrophobic, and their main limitation is the low aqueous solubility, which leads to poor bioavailability and difficult application. Encapsulation of these substances into hydrophilic nanoparticles, such as nanogels, could significantly improve their characteristics [116]. For example, encapsulation of a hydrophobic polyphenol curcumin into a biopolymeric nanogel system based on acylated rapeseed protein isolate increased curcumin solubility and cytotoxic activity in vitro [117]. The main mechanism of loading hydrophobic drugs in nanogels was expected to be hydrophobic interactions with the respective domains of amphiphilic polymeric carriers of nanogels. Curcumin was loaded into nanogel particles based on N-isopropylacrylamide and N,N-dimethylaminoethylmethacrylate (DMAEMA) co-monomers graft-copolymerized from fully brominated lignin [118]. The hydrophobicity of lignin determined a high loading due to hydrophobic interactions and some delay in the release process. On the other hand, the pH-dependent behavior of DMAEMA units (tertiary amine functional groups deprotonated at basic pH and protonated at acidic pH) was responsible for the degradation of nanogel particles and drug diffusion. The polyphenol resveratrol has been embedded via hydrophobic interactions into biocompatible nanogel particles based on citric acid and pentane-1,2,5-triol with an encapsulation efficiency of 94.5% [119]. The entrapment of resveratrol into the nanogel provided its complete release for 24 h, whereas the release of pure resveratrol for the same period was incomplete (only 11% for 24 h). Thus, the encapsulation of resveratrol into the nanogel system improved its biopharmaceutical behavior. Paclitaxel, a hydrophobic taxane, has been loaded via hydrophobic interactions into a nanogel composed of cholesterol-grafted methacrylated hyaluronic acid copolymer [31]. The nanogel particles were functionalized with biotin that additionally provided biotin receptor-mediated cellular uptake. The release of paclitaxel was a result of pH- and enzyme-triggered (hyaluronidase and/or lipase) degradation of the nanogel structure. Double loading of paclitaxel and quercetin in folic acid–gelatin–Pluronic P123 nanogels was achieved through electrostatic and hydrophobic interactions [19]. The poly(ethylene oxide) chains of Pluronic P123 participated in hydrophobic interactions with the drug molecules while gelatin formed electrostatic bonds. Interestingly, the loading of both drugs into the nanogel particles was significantly higher than that in Pluronic P123 micelles. The release mechanisms were Fickian diffusion for quercetin and non-Fickian diffusion for paclitaxel. In vitro tests on human breast MCF-7 and cervical cancer HeLa cells, and in vivo on MCF-7 tumor-bearing mice, showed that the folic acid-targeted nanogels improved drug cytotoxicity and reduced side effects. The essential oil from *Mentha piperita* has been loaded into chitosan nanogel particles via physical incorporation into the pores of the matrix and electrostatic interactions. The oil was released depending on the swelling and the degradation of the nanogel matrix [51].

The hydrophobic drug β-lapachone was loaded via hydrophobic interactions in a nanogel based on poly-N-isopropyl acrylamide co-polymerized with acrylic acid [120]. Due to the properties of the copolymer, the release of the drug was controlled by the temperature and pH of the medium. Two drugs, olaparib (inhibitor of poly(ADP-ribose) polymerase) and doxorubicin, were co-loaded in poly(ethylene glycol)-poly(L-phenylalanine-co-L-cystine) nanogels containing disulfide bonds [121]. The drugs were loaded by the nanoprecipitation technique, reaching 51% and 23% drug loading efficiency for doxorubicin and olaparib, respectively. The authors reported that the drugs were inserted into the hydrophobic core, and the efficiency for the more hydrophobic olaparib was lower. The release of both drugs was significantly faster in phosphate buffer containing glutathione, which indicated that the release process was governed by a cleavage of the disulfide bonds.

Except for hydrophobic interactions, other types of interactions could also contribute to loading of hydrophobic drugs. For example, quercetin was loaded into sodium alginate nanogel particles through hydrogen bonding between the hydroxyl groups of alginate and quercetin [122]. Curcumin was encapsulated in poly(N-isopropyl acrylamide)-chitosan nanogels co-loaded with gold nanoparticles [123]. The authors stated that the loading of curcumin occurred by two types of interactions, particularly (1) hydrophobic interactions with PNIPAM segments and (2) conjugation of curcumin to the gold nanoparticles. The hydrophobic drug benzophenone was successfully loaded in nanogel via association of a hydrophobically modified dextran (bearing dodecyl groups) and a β-cyclodextrin [124]. The loading of the drug occurred in two ways: (1) inclusion of the drug in cyclodextrin cavities due to Van der Waals forces, and in a lower degree (2) solubilization of the drug into the hydrophobic microdomains of the amphiphilic modified dextran. Since the drug was more efficiently loaded in the cavities of β-cyclodextrin in the associative nanogel, the release process was driven by the dissociation of these complexes. The complexation strategy was also applied for loading resveratrol into a composite nanogel prepared from bovine serum albumin and chitosan [125]. In this study, resveratrol was included in a hydroxypropyl-β-cyclodextrin complex that further was encapsulated into the nanogel, achieving 98% encapsulation efficiency. In another study, aldehyde-modified β-cyclodextrin and hydrazide-modified carboxymethylcellulose were used for the development of a nanogel platform for ginsenoside CK delivery [126]. The hydrophobic drug was loaded into the hydrophobic cavity of the cyclodextrin with high efficiency. The drug release occurred by a combination of drug diffusion through the polymer and dissolution of the polymer. The process was enabled in an acidic medium since the hydrazide and the aldehyde groups influenced the structure of the nanogel in such a medium.

**Table 5:** Nanogels loaded with hydrophobic substances.

Composition of Nanogels	Active Substance	Mechanism of Release	Encapsulation Efficiency (%)	Reference
Carboxymethyl starch–chitosan hydrochloride	Curcumin	pH-dependent swelling and diffusion	89–94	[48]
Hydroxypropyl-β-cyclodextrin, bovine serum albumin and chitosan	Resveratrol	First order kinetics diffusion	97.8	[125]
Aldehyde-modified β-cyclodextrin and hydrazide-modified carboxymethylcellulose	Ginsenoside CK	pH-dependent drug diffusion and polymer dissolution	70.9	[126]
Chitosan and ρ-coumaric acid	*Lippia origanoides* essential oil	n.d.	42	[127]
Pectin and lysozyme	Methotrexate	Drug diffusion and pH-responsive swelling/degradation	58.6	[62]
Cholesterol-grafted methacrylated hyaluronic acid and biotin	Paclitaxel	Enzyme-induced degradation of nanogels	90.2	[31]
Folic acid-gelatin-Pluronic P123	Quercetin and paclitaxel	Diffusion	88.6 (quercetin) 98.7 (paclitaxel)	[19]
Chitosan	*Mentha piperita* essential oil	Swelling and degradation of nanogel	n.d.	[51]
Chitosan	Glibenclamide and quercetin	Degradation of nanogel	94.2 (glibenclamide) 97.5 (quercetin)	[20]
Albumin and carboxymethyl cellulose	Camptothecin	pH-dependent	90	[66]

## 6. Challenges of Different Routes of Administration

### 6.1. Parenteral Route

Among different parenteral routes of application, i.v. administration is the most frequently applied. The main limitation of such administration is opsonization and further phagocytosis of the injected nanogels. The result is fast elimination from the blood circulation and a poor delivery to the desired site in vivo. Thus, independent of the presence of targeting ligands, the drug-loaded nanogels could not reach the selected tissues and cells. Two properties of the nanogels are very attractive, aiming to overcome the rapid elimination. First, nanogels are hydrophilic nanostructures, which makes opsonization more difficult. Second, the formation of an outer corona from poly(ethylene glycol) would ensure the “stealth” properties of nanogel particles. It is very important to note that “stealth” properties are closely related to the texture of the nanogel system, the flexibility of chains, and the density of PEG shell. Anselmo et al. have reported that softer PEG-based hydrogel nanoparticles (with elastic moduli of 10 kPa) prolonged circulation time in vivo more efficiently than harder nanoparticles (3000 kPa) [128]. It is important to emphasize that PEG shell also hinders aggregation between nanogel particles because of steric phenomenon. Thus, prevention of aggregation ensures that the small size of particles is maintained, which is a key factor for avoidance of phagocytes. Other hydrophilic polymers could also be considered attractive as a “stealth” outer shell of nanogels. Some studies reported that dextran shell could provide steric protection against protein adsorption. Nanogel particles composed of a dextran shell and lysozyme core possessed stealth properties in vitro in a model of differentiated macrophages (phorbol 12-myristate 13-acetate stimulated human monocyte-derived macrophage cell line THP-1) [129]. Flow cytometry and confocal microscopy indicated slow uptake of the developed nanogels into the stimulated THP-1 cells, particularly 20% and 41% at 3 h and 24 h incubation time, respectively. Polysarcosine, a polypeptide derived from the N-methylated derivative of glycine, is hydrophilic and possesses a neutral surface charge. It is an appropriate alternative for the development of stealth nanogels, taking into consideration some studies reporting more prolonged blood circulation of polysarcosine-modified nanoparticles compared to similar PEG-modified nanoparticles [130].

**Figure 4:**
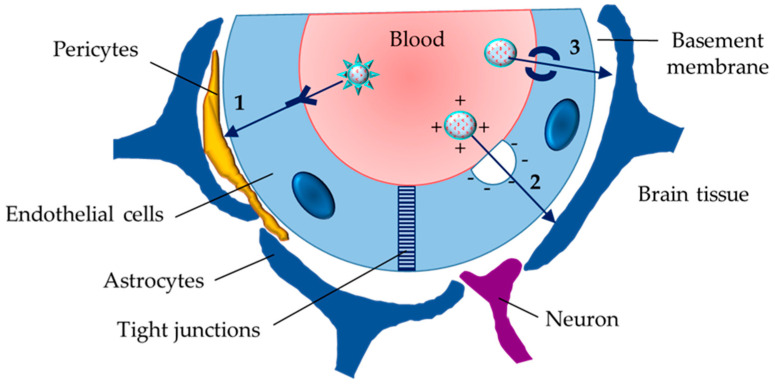
Schematic presentation of the different mechanisms for nanogel transport through a blood–brain barrier: (1) receptor-mediated transcytosis, (2) adsorption-mediated transcytosis, and (3) transporter-mediated transcytosis.

The i.v. administered nanogels could be intended to deliver therapeutic agents to brain tissue. However, the transport through the blood–brain barrier (BBB) is highly restricted. The barrier includes endothelial cells that form the walls of the blood vessels, pericytes, astrocytes, neurons, and basement membrane. The brain endothelial cells have different properties compared to endothelial cells of other tissues and they act as an integrated structure with the other cells (Figure 4). Furthermore, the endothelial cells are bonded by tight junctions formed by transmembrane protein complexes of occludin, claudin, and junction adhesion molecules (members of the immunoglobulin superfamily). These features strongly restrict paracellular diffusion of solutes. Thus, transcellular transport is a more realistic process. It could be achieved by different mechanisms, in particular receptor-mediated, adsorption-mediated (for positively charged solutes), or transporter-mediated transcytosis (Figure 4). The main parameters of nanogels that play a role in the occurrence of transcellular transport are particle size, hydrophilicity, and softness. In this view, it was indicated that the particle size of drug delivery systems should be between 50 and 200 nm to ensure their deposition in the brain [131]. Kimura et al. have reported that gelatin nanogels with a particle size of 5–21 nm successfully entered the brain parenchyma through the BBB after i.v. injection [132]. After i.v. administration of chitosan-based nanogel particles loaded with methotrexate (118 nm), the concentration of the drug in the rat brain was significantly higher (more than 10-fold) compared to that of injected non-encapsulated drug [133]. The authors postulated that the small size, cationic charge, and hydrophilic properties were the factors that determined the transport of the drug-loaded nanogels across the BBB. The surface modification of the developed nanogels with polysorbate 80 did not provide more effective passing through the barrier.

Importantly, the combination of small size and targeting ligands could additionally enhance the effectiveness of transport through the barrier. The brain capillary endothelial cells highly express transferrin receptors that could be exploited as a potential target for brain drug delivery. Vinogradov et al. designed poly(ethylene glycol)-polyethylenimine nanogel particles (size less than 100 nm) modified with transferrin or insulin [105]. The nanogel particles demonstrated successful transcellular transport across the bovine brain microvessel endothelial cells used as a model of the blood–brain barrier. In vivo mouse model revealed that 1 h after intravenous injection, the accumulation of encapsulated oligonucleotides in the brain increased by over 15-fold compared to the non-encapsulated oligonucleotides. Lactoferrin is a glycoprotein (possessing two transferrin families) that could be specifically bound by low-density lipoprotein receptor-related protein 1 (LRP-1), which is highly expressed on BBB endothelial cells and glioma cells. Thus, lactoferrin was attached as a targeting ligand to nanogels constructed via cross-linking of phenylboronic acid-modified hyaluronic acid with bis(methacryloyl) cystamine [112]. Significantly improved brain permeability in vitro and brain accumulation in vivo after i.v. administration to rats confirmed the role of lactoferrin ligands for better transport through the BBB. Similarly, Angiopep2 is a peptide that could be considered as a targeting ligand because it binds specifically to LRP-1 [134]. Nanogel particles based on carboxymethyl chitosan cross-linked with cystamine were modified with Angiopep2, aiming to improve its transport through the BBB [135]. The targeted nanogel showed significantly higher uptake in vitro and in vivo (in mice) compared to the non-targeted formulation. In addition, the dual-responsivity of the nanogel particles enabled drug release intracellularly. In particular, both the acidic pH and the high concentration of glutathione, which disrupted the disulfide bonds and destroyed the cross-linking structure, ensured the release of the incorporated doxorubicin.

Another study revealed that the soft and deformable nature of nanogels could also contribute to transportability. Soft nanogels demonstrated a 2-fold higher secretion at the basal side of the BBB model compared to stiff nanogels, probably due to enabled transcytosis [136]. The similarity between the nanogel carrier and cellular membrane also has a crucial role. A study showed that zwitterionic phosphorylcholine nanogels passed through the BBB and delivered the loaded drug in glioblastoma tissue due to the phosphorylcholine mimicking cellular membrane [137].

### 6.2. Oral Route

Oral drug administration is the most preferred one since it is non-invasive and easy for patients, which is a prerequisite for good compliance. The structure of the gastrointestinal barrier includes epithelial cells that are coated with a mucus layer consisting of adherent and loose sublayers [43]. Thus, the gastrointestinal barrier could hinder the absorption of drug molecules since there are various factors such as the thickness and composition of mucus, different pH of the fluids in the tract, various enzymes, microbial flora, and hepatic metabolism. Incorporation of drugs into nanoparticles is an approach to protect them from metabolism, to inhibit different efflux pumps, and to enhance absorption due to small mean diameters and enhanced aqueous solubility [138].

**Figure 5:**
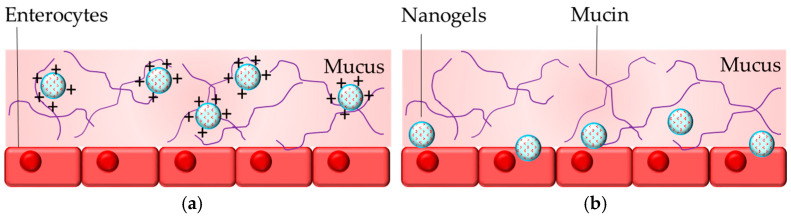
Schematic presentation of mucoadhesive (**a**) and mucopenetrating nanogels (**b**) in the gastrointestinal mucus layer.

Nanogel drug delivery systems are particularly suitable for transmucosal delivery since they are hydrophilic and can be modified aiming to provide mucoadhesive or mucopenetrating properties. The mucoadhesive properties of the formulations will provide retention of the loaded drug in the loose mucus layer. The mucopenetration will ensure the entrance of the drug into the adherent mucus layer and close interactions with the epithelial cells. Briefly, positively charged nanogels (e.g., obtained from chitosan) mainly enhance the delivery of substances via mucoadhesion, while the neutral ones increase the mucopenetration (Figure 5). Moreover, some of the polymers (e.g., poly(acrylic), poly(methacrylic) acid, chitosan, etc.) could additionally provide pH-dependent swelling and drug release.

Importantly, the encapsulation of labile drugs into nanogels could protect them from the inappropriate pH of gastric fluids and enzymatic activity. For example, Wang et al. have loaded insulin into a hydroxyethyl methacrylate nanogel prepared via the emulsion polymerization method [139]. The encapsulated insulin extended the hypoglycemic effect to 12 h after oral administration of the nanogel particles in rats. Furthermore, the overall bioavailability of the encapsulated insulin was much higher than that of the pure insulin. Thus, the softness, nanosize, and swelling capacity were considered as main factors for the intestinal absorption. The second reason was that the nanogel particles protected insulin from degradation in the gastric fluid. A novel nanogel–polymersomes drug delivery system was developed from chitosan diacetate, methoxy-poly(ethylene glycol)-b-poly (lactide) and D-alpha-tocopheryl polyethylene glycol succinate [140]. The system was intended for oral administration and was double loaded with oxaliplatin and rapamycin. The stability of both drugs in medium with a pH of 1.2 was significantly improved, and their release in intestinal segments was achieved. Chitosan-g-poly(methyl methacrylate) nanogels thiolated with N-acetyl cysteine have been tested in vitro for mucoadhesive and mucopenetrating properties [141]. Two models of intestinal barrier were used in the experiment, namely a monolayer colon Caco-2 cells and a co-culture of Caco-2 cells with mucous-secreting colon HT29-MTX cells. There was no difference between the permeability of unmodified and thiolated nanoparticles in the monolayer of Caco-2 cells. However, the thiolated nanogels showed diminished permeability through the co-culture cells, probably due to interactions with the cysteine domains of the mucin. This study showed that depending on the nanogel’s modification, a desired pattern of penetration could be obtained.

### 6.3. Nasal Route

The nasal route of administration gains more and more popularity since it possesses various advantages, which provide increased bioavailability of drugs, resulting in improved pharmacological effects. For instance, the drug molecules are protected from the acidic pH of the stomach and first-pass metabolism. Transport through the nasal mucosa is realized via intracellular (endocytosis) or paracellular transport. This route of administration could provide a local or systemic effect of drugs, and it is a direct pathway to the brain. It is also an appropriate route for vaccine delivery since the mucosa is rich in immune fragments. However, the nasal mucosa is covered with ciliated epithelium and mucus, which is related to the elimination of the drugs. Also, there are enzymes that could metabolize some drug molecules and hinder their therapeutic activity. Thus, nanogels could protect drugs from the external medium (including enzymes) and could provide mucoadhesion/mucopenetration. The nasal permeation of propranolol has been enhanced by its formulation in limonene-based microemulsion and further encapsulation into nanogel prepared from Poloxamer-407 and chitosan [142]. First, the thermo-responsive nature of Poloxamer (fast sol–gel transition above 30 °C) and the mucoadhesive properties of chitosan prevented the outflow of the nanogel particles through the mucociliary clearance. Second, the lipophilic properties of the microemulsion as well as the presence of limonene (a terpene penetration enhancer) also contributed to the enhanced permeation. In addition, the small size of the nanogel vehicles increased the surface area available for propranolol permeation through nasal mucosa. Nanogel from dextran sulfate and poly-L-lysine was obtained for nasal delivery of ovalbumin as a model antigen [28]. The system was characterized by positive surface charge, which mediated the interaction with the mucin and provided sustained release of the protein. Pullulan is another polysaccharide with well-pronounced bioadhesive properties that could provide longer residence of nanogels on nasal mucosa. For example, a non-toxic subunit fragment of *Clostridium botulinum* type-A neurotoxin BoHc/A has been encapsulated into cationic cholesteryl-group-bearing pullulan nanogel [61]. The formulation showed prolonged retention in the nasal mucosa in mice (more than two days) in comparison with the free neurotoxin (retained for only 6 h). Cationic cholesteryl pullulan nanogel was also developed for nasal delivery of trivalent pneumococcal surface protein A vaccine for the treatment of pneumococcal infections [143]. The protein-loaded nanogel effectively induced a specific IgG in macaques for binding *Streptococcus pneumoniae* and triggered a complement C3 deposition, resulting in a high bactericidal effect. As mentioned above, the intranasal delivery could be considered a direct route for drug administration to the brain, bypassing the blood–brain barrier. Insulin has been attached via covalent bonds with poly(N-vinyl pyrrolidone)-co-acrylic nanogel particles for nose-to-brain delivery for treatment of diseases such as Alzheimer disease [144]. In vivo experiments in mice showed that the formulation (92 nm) did not induce morphological alterations and immunological response in nasal mucosa and brain, which means that it is well-tolerated. The nanogel particles improved the transport of insulin to the mouse brain since a higher concentration was detected after their administration compared to free insulin. The improved transport was probably reliable on the mucoadhesive properties of the nanogel particles, enhanced permeation through the tight junctions since the carboxyl groups on the nanogel surface could chelate calcium ions in the membrane, and the protection of the insulin from proteolytic enzymes.

### 6.4. Ocular Route

The non-invasive ocular route of administration is used for local delivery of drugs for treatment of different ocular diseases. However, there are some aspects, such as the intense lacrimal drainage and the corneal barrier, which hinder the therapeutic action of pharmaceuticals. Some nanogels could improve the pharmacological effects of drugs via enhanced mucoadhesion, prolonged retention time, and mucopenetration. The cornea consists of endothelium, Descemet’s membrane, stroma, Bowman’s layer, and epithelium, and it is covered with a mucus layer. The epithelium interferes with the transport of hydrophilic substances, while the stroma hinders the absorption of lipophilic drugs. Amphiphilic nanogels could overcome these obstacles and improve the efficacy of therapies. Hyaluronic acid–cystamine–cholesterol nanogel, complexed with the cell-penetrating peptide penetratin, has been obtained and cross-linked via thiol–disulfide bonds for topical application in the posterior part of the eye [145]. In vitro release tests of fluorescin-loaded nanogel showed redox-triggered release due to breaking of thiol–disulfide bonds between the molecules of hyaluronic acid in the presence of high concentrations of 1,4-dithiothreitol. This could lead to targeted delivery into a reductive environment in the cytoplasm. The visual chromophore chemical analog, 9-cis-retinal, has been loaded into the same nanogel. In vitro cellular uptake in retinal pigment epithelia ARPE-19 cells was improved, especially when the formulation was modified with penetratin. In vivo tests showed that the loaded nanogel led to the regeneration of retinal pigmented epithelium cells. Probably, the hyaluronic acid-mediated binding with retinal pigmented epithelium CD44 receptor and the improved hydrophilicity of the substances increased the effect. Amphotericin B has been loaded into alkyl glyceryl hyaluronic acid nanogel for ophthalmic delivery [32]. The mucoadhesive properties of the formulation have been proved since there was an interaction between the polymer and mucin, leading to a decrease in the zeta potential and increased adhesion to the mucin-bound nitrocellulose membrane. This combination with the small size of the nanogel particles (31.5 nm) could lead to prolonged corneal retention in vivo. The hydrophobic drugs dexamethasone and piroxicam and the hydrophilic tobramycin and diclofenac sodium salt have been loaded into hyaluronan–cholesterol nanogels [60]. Due to the bioadhesive properties of the hyaluronic acid, the authors observed prolonged contact time with porcine cornea. Furthermore, the capability of hyaluronic acid to interact with CD44 receptors and the ability of the hydrophobic cholesterol units to interact with the components of corneal epithelium enhanced the permeability. However, this formulation was considered to be more appropriate for delivery of hydrophilic drugs since the transport of the hydrophobic drugs through the stroma was still hindered due to their high lipophilicity. The permeation ability of dexamethasone (vs. market product Luxazone), tobramycin (vs. non-loaded drug), and diclofenac (vs. non-loaded drug) has been enhanced; however, the ability of piroxicam (log P = 3.06) was lower than the commercial product Piroftal, probably due to the presence of viscosity-enhancing polymers in the market product.

### 6.5. Dermal Route

**Figure 6:**
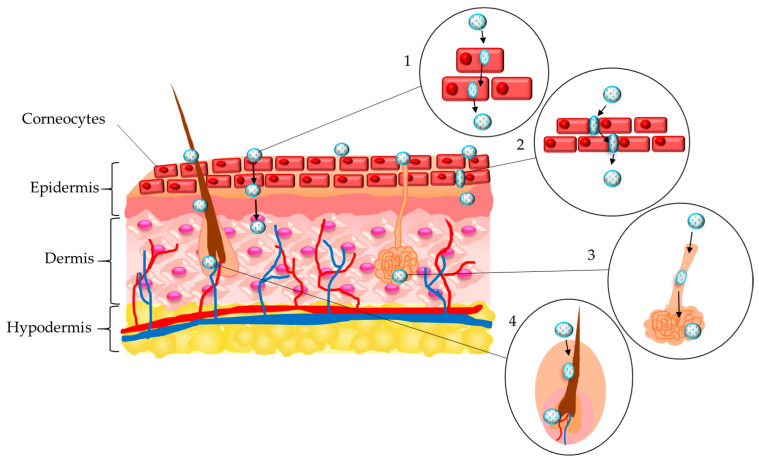
Schematic presentation of the different mechanisms for nanogel transport through the skin: (1) transcellular, (2) intercellular, (3) through sweat glands, and (4) through hair follicles.

Skin is the largest organ in the human body, and it consists of three layers—epidermis, dermis, and hypodermis. The epidermis is the outer layer, and it has a function of a chemical and physical barrier of the body. The outer part of the epidermis, the stratum corneum, is a horn-like layer consisting of dead keratinocytes. This layer is considered to be the main limiting barrier for hydrophilic substances and drug-loaded nanoparticles since it prevents their absorption. Some lipophilic substances also could be retained from epidermis, which hinders their therapeutic effect. There are two routes of penetration of drugs and nanoparticles through the skin—transepidermal (transcellular and intercellular) and transappendageal (through sweat glands and hair follicles) (Figure 6). The transcellular route is suitable for hydrophilic substances. Usually, this route requires a penetration enhancer. The intercellular route is used for uncharged lipophilic molecules since the space between the keratinocytes is filled with lipophilic compounds. The transappendageal routes are the shortest ones, and penetration through hair follicles could provide a systemic effect of drug-loaded nanoparticles because of the large amount of blood vessels and lymph nodes. However, the rate of transappendageal penetration and the efficacy of the process depend on the content and amount of secretion and the frequency of glands and hair follicles [146]. Nanogels could enhance the permeation of drugs through the epidermis since they are hydrophilic and could moisturize this outer layer, possess a small mean diameter, and have a deformable shape. For example, terbinafine hydrochloride has been encapsulated into pH-responsive Pluronic F127 co-poly(acrylic acid) nanogel for treatment of skin fungal infections [75]. The nanogel provided a faster release in medium with pH 7.4 that was considered appropriate for treatment of the fungal infection site. The formulation showed improved retention in rat skin ex vivo, namely approximately 42% of the drug, in comparison with the commercial product 1% Lamisil (approximately 27% retention). This phenomenon was explained with the hydrophilic nature of the nanogel that increased the hydration of the stratum corneum and enabled the formation of channels for enhanced skin penetration. Furthermore, the highly elastic and deformable properties of nanogel also contributed to the more effective retention compared to the commercial product. Rajput et al. loaded the antifungal agent luliconazole into pH-responsive poly(acrylic acid)-sodium carboxymethyl cellulose nanogel for targeted treatment of skin infections [99]. The permeation through goat pinna skin was enhanced in comparison with that of the market product Lulixol. Furthermore, ex vivo drug release was increased due to the glycoproteins present in the skin. Lee et al. examined the possibility of achieving transcutaneous antigen delivery by loading it in pectin nanogel [147]. The authors developed thiol-cross-linked nanogels from norbornene-functionalized pectin with loading of thiolated ovalbumin. The nanogel particles loaded with ovalbumin penetrated through the stratum corneum and were deposited in the epidermis and dermis, while the non-encapsulated ovalbumin did not penetrate the stratum corneum. Chitin nanogels were prepared as delivery systems for two anti-psoriatic drugs, acitretin and aloe–emodin [148]. Skin permeation studies through porcine skin showed higher deposition of the nanogels at epidermal and dermal layers. The authors discussed that the main reasons for the enhanced permeability are the small size of the nanogels (138 nm and 238 nm for acitretin and aloe–emodin nanogels, respectively), the hydrophobicity of the polymer, and the positive charge of the systems. Gerecke et al. reported that thermo-responsive nanogels synthesized from dendritic polyglycerol with poly(glycidyl methyl ether-co-ethyl glycidyl ether) were capable of enhancing penetration through biological barriers such as the stratum corneum and were taken up by keratinocytes of human skin without cytotoxic or genotoxic effect [149].

Nanogels are very appropriate for wound healing applications due to enhanced moisturizing and retention of drugs in the skin. For example, the encapsulation of prostaglandin E1 in cholesterol-bearing pullulan nanogel improved its wound healing ability, since the formulation protected it from oxidation and inactivation and provided prolonged delivery. This nanogel contained a large amount of water, and it could be exchanged for exudates, which would keep the place in constant moisture [150]. The entrapment of the antioxidant baicalin in gellan–cholesterol nanogel led to complete wound healing, inhibition of tumor necrosis factor-α, and edema in 2-O-tetradecanoylphorbol 13-acetate-injured skin of mice [151]. The nanogel enhanced the moisturizing of the skin and penetration of baicalin in comparison with commercial cream containing betamethasone. Polypeptide nanogels with or without silver nanoparticles showed good wound healing effects, resulting from their antimicrobial and anti-inflammatory activity, comparable to standard hydrogels [152].

## 7. Closing Remarks and Perspectives

Nanogels are advantageous drug delivery systems capable of overcoming some of the obstacles related to the different physiological membranes. The great variety of non-toxic natural polymeric carriers as well as the mild conditions for preparation give nanogels an important role as sustainable drug delivery systems. The perspectives are related to the development and characterization of nanogels that combine different non-toxic carriers capable of providing high drug loading and stimuli-responsive delivery in in vivo conditions.

## Figures and Tables

**Table 1:** Examples of nanogels prepared from natural polymers and their advantages.

Polymer	Loaded Drug and Advantages of the System	Reference
Cinnamic acid-grafted chitosan	Essential oils of *Syzygium aromaticum* and *Cinnamomum ssp.* Complete inhibition of *Microsporum canis* growth	[25]
Chitosan	Thymol Improved antibacterial activity	[26]
Chitosan	Trinitroglycerin Accelerated wound healing	[27]
Dextran sulfate and ε-polylysine	Ovalbumin Sustained release	[28]
L-arginine-modified dextran	Ovalbumin Controlled release, prolonged antigen storage Enhanced inducement of cellular and humoral immunity	[29]
Soybean protein isolate-dextran	CurcuminEnhanced antioxidant activity and stability	[30]
Biotin-functionalized cholesterol-grafted methacrylated hyaluronic acid	Paclitaxel Stimuli-responsive release Improved pharmacokinetic profile Enhanced antitumor effects in vivo	[31]
Alkyl glyceryl hyaluronic acid	Amphotericin B	[32]
	Mucin interactions, lowered toxicity Enhanced fungal selectivity	
Histidin-modified methacrylated hyaluronic acid	Vatalanib and near-infrared photothermal agent ICG Targeted release Inhibition of tumor angiogenesis Enhanced antitumor effect	[33]
Pectin and alginate	Naringin Improved storage stability	[34]
Bovine serum albumin and gum arabic aldehyde	5-fluorouracil pH-sensitive release	[35]
Bovine serum albumin and chitosan	Doxorubicin Improved photostability Enhanced anticancer effect	[36]
Ovalbumin and pullulan	Curcumin Controlled release	[37]
Glutathione-responsive methylated poly(ethylene glycol)-poly (phenylalanine)-poly(cystine) block copolymer	Shikonin Redox-sensitive release Targeted tumor biodistribution Enhanced anticancer effect	[38]
Carboxymethyl chitosan lysozyme	Tea polyphenols, silver nitrate, or chlorhexidine Inhibitory effects against *Streptococcus mutans* Good remineralization of early enamel caries surface	[39]

**Table 2:** Examples from the last five years of nanogels prepared from synthetic polymers and their advantages.

Polymer	Loaded Drugs and Advantages of the System	Reference
Pluronic F127 co-poly(acrylic acid)	Terbinafine HCL Increased dermal retention Enhanced antifungal effect in vitro and in vivo	[75]
Poly(acrylic) acid	Mitoxantrone and metallic manganese ions pH-dependent release Enhanced anticancer effect in vitro and in vivo	[76]
Poly(acrylic acid)	Bombesin derivate peptides and radiolabeling Increased internalization in adenocarcinoma cells	[77]
Glutathione-conjugated poly(methacrylic acid)	Edaravone Sustained release Elevated spatial memory, learning, and cognitive function in rats	[78]
Poly(ethylene glycol)-poly(methacrylic acid)	Olmesartan Increased solubility and pH-dependent release	[79]
Poly(methacrylic acid)-block-poly(3-fluorophenylboronic acid methacrylamide)	Cisplatin Achieved cell death	[80]
Polyethylenimine	Docetaxel and CD47 siRNA Redox-responsive release and targeted delivery	[81]
Pluronic P123-polyethylenimine	Doxorubicin Successful coating of hydroxyapatite nanoparticles pH-responsive release and enhanced anticancer effect	[82]
Low-molecular-weight poly(ethylenimine)-poly(ethylene glycol)	Transforming growth factor-β1 siRNA and ultrasmall iron oxide nanoparticles Enhanced magnetic resonance imaging performance Enhanced antitumor effect in vitro and in vivo	[83]

**Table 3:** Examples of nanogels based on a combination of natural and synthetic polymers.

Polymer	Loaded Drugs and Advantages of the System	Reference
Hyaluronic acid and polyethylenimine	Doxorubicin Sustained release Enhanced cell internalization	[94]
Poly(acrylic acid) and deoxycholic acid-modified chitosan	Insulin Increased stability of the hormone	[95]
Pullulan and poly(acrylic acid)	Doxorubicin pH-dependent release Enhanced cytotoxicity Alleviated adverse effects in vitro	[96]
Pectin and poly(acrylic acid)	Rutin pH-responsive release Enhanced skin permeation Enhanced anticancer effect in vitro	[97]
